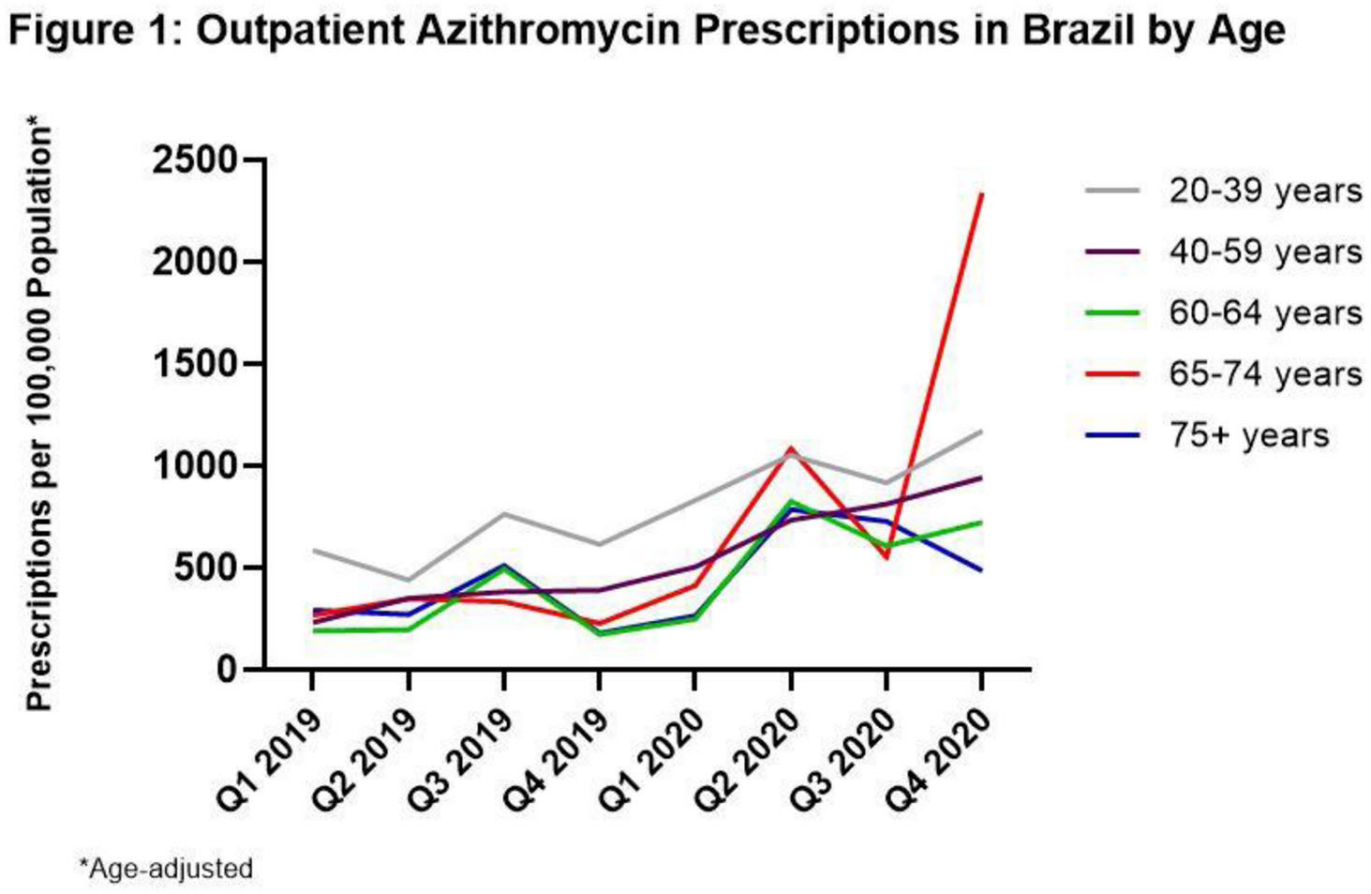# Prescribing of common outpatient antibiotics for respiratory infections in adults amid the COVID-19 pandemic in Brazil

**DOI:** 10.1017/ash.2022.149

**Published:** 2022-05-16

**Authors:** Dipesh Solanky, Olivia McGovern, Fernanda Lessa, Lauri Hicks, Sharon Tsay, Payal Patel

## Abstract

**Background:** Inappropriate antibiotic use for SARS-CoV-2 infection has the potential to increase the burden of antibiotic resistance. Brazil experienced spread of a new SARS-CoV-2 variant in the fourth quarter (Q4) of 2020, resulting in the highest case counts in Latin America, raising concerns of antibiotic overuse. To better understand antibiotic use during the COVID-19 pandemic, we evaluated prescribing changes in antibiotics commonly used for outpatient respiratory infections (amoxicillin-clavulanate, azithromycin, and levofloxacin or moxifloxacin [AALM]) among adults aged ≥20 years in Brazil in 2020 versus 2019. **Methods:** We analyzed the IQVIA MIDAS medical data set for AALM prescribing by age group (20–39, 40–59, 60–64, 65–74, ≥75 years), comparing Q4 2020 rates to those in Q4 2019. We estimated crude rate ratios and 95% CIs using prescription number as the numerator (assuming Poisson counts) and age-adjusted population as the denominator. We also determined the most common prescribing specialties for each antibiotic across both time points. **Results:** Compared to Q4 2019, Q4 2020 azithromycin prescribing increased among all ages, ranging from 90.7% (95% CI, 90.0%–91.4%) in those aged 20–39 years to 927.2% (95% CI, 912.9%–941.7%) in those aged 65–74 years (Fig. 1). Amoxicillin-clavulanate prescribing decreased for most ages, ranging from −78.4% (95% CI, −78.7% to −78.1%) in those aged 60–64 years to −25.8% (95% CI, −26.6% to −25.0%) in those aged 65–74 years. Prescribing of levofloxacin or moxifloxacin decreased for most ages, ranging from −39.1% (95% CI, −39.4% to −38.8%) in those aged 20–39 years to −16.9% (95% CI, −18.1% to −15.7%) in those aged 60–64 years. For those aged ≥75 years, prescribing of amoxicillin-clavulanate and levofloxacin or moxifloxacin increased by 13.2% (95% CI, 11.9%–14.5%) and 43.1% (95% CI, 41.7%–44.5%), respectively. In Q4 2019 and Q4 2020, the 2 most common prescribing specialties for azithromycin were general practice (48%–50% of prescriptions) and gynecology (19%–25%). Compared to Q4 2019, infectious disease specialists in Q4 2020 saw the largest decline in percentage of azithromycin prescriptions (10% to 1%) and surgeons saw the largest increase (0% to 7%). General practitioners were also the most common prescribers of the remaining antibiotics (43%–54%), followed by gynecology for levofloxacin or moxifloxacin (25%–29%) and otolaryngology for amoxicillin-clavulanate (14%–20%). **Conclusions:** Despite decreases in prescribing of amoxicillin-clavulanate and respiratory fluoroquinolones for most adults, azithromycin prescribing increased dramatically across all adults during the COVID-19 pandemic. Targeting inappropriate outpatient antibiotic use in Brazil, particularly azithromycin prescribing among general practitioners, gynecologists, and surgeons, may be high-yield targets for antibiotic stewardship.

**Funding:** None

**Disclosures:** None

**Figure:**